# Developing a Suite of Motion-Controlled Games for Upper Extremity Training in Children with Cerebral Palsy: A Proof-of-Concept Study

**DOI:** 10.1089/g4h.2017.0141

**Published:** 2018-11-14

**Authors:** Jen-Wen Hung, Yao-Jen Chang, Chiung-Xia Chou, Wen-Chi Wu, Stephen Howell, Wei-Peng Lu

**Affiliations:** ^1^ Department of Rehabilitation, Chang Gung Memorial Hospital-Kaohsiung Medical Center, Kaohsiung, Taiwan.; ^2^ School of Physical Therapy, College of Medicine, Chang Gung University, Taoyuan, Taiwan.; ^3^ Department of Electronic Engineering, Chung Yuan Christian University, Chung-Li, Taiwan.; ^4^ SMARTlab, University College Dublin, Dublin, Ireland.

**Keywords:** Cerebral palsy, Kinect, Scratch, Rehabilitation, Upper extremity

## Abstract

***Aim:*** The Scratch programming language allows learner developers to write games. The Kinect2Scratch extension makes Scratch games with bodily motion control possible by connecting to Microsoft's Kinect sensor. This study examined the feasibility and possible efficacy of a suite of motion-controlled games designed for upper extremity (UE) training in children with cerebral palsy (CP) using Kinect2Scratch.

***Materials and Methods:*** This is a proof-of-concept study. We developed three games, requiring three UE movement patterns (shoulder holding, reaching, and handclap), for use in children with CP. The primary outcome was feasibility, addressed by adherence, engagement, satisfaction, and safety. The secondary outcome was efficacy, which was evaluated by Quality of Upper Extremities Skills Test (QUEST), Box and Block Test (BBT), Melbourne Assessment 2 (MA2) test, and ABILHAND-kids score.

***Results:*** Thirteen children with CP (mean age 6.9 years) received 24 sessions of training (30 minutes per session). The adherence rate was 100%. During the first 2 weeks of training, children had a significantly higher level of participation in Kinect2Scratch training than in conventional rehabilitation [Pittsburgh Participation Scale, median (interquartile range [IQR]), 6 (3–6) vs. 4 (3–6) *P* = 0.04]. However, during the last 2 weeks of training, there was no significant difference in participation between the Kinect2Scratch and conventional training [Pittsburgh Rehabilitation Participation Scale, median (IQR), 4 (3–5) vs. 4 (3–6) *P* = 0.55]. Most children enjoyed playing the games. The mean score of enjoyment was 4.54 ± 0.66. There were no adverse events during the training periods. The children had significant improvement in total score of QUEST and MA2. There were no significant improvements in BBT and ABILHAND-kids score.

***Conclusion:*** Using Kinect2Scratch games for UE training is a feasible adjunctive program for children with CP.

## Introduction

Cerebral palsy (CP) is the most common cause of physical disability in childhood with more than 2 cases per 1000 live births.^1^ Children with CP usually have various functional impairments of their upper extremities (UEs), which might reduce their independence in activities of daily living (ADL). Function training for the UEs is an important issue in CP rehabilitation.

Current evidence supports that goal-directed, task-specific, and high-dose repetition therapy could lead to better motor outcomes.^2–7^ However, the intensive repetitive training has been a challenging task for pediatric rehabilitation, because such therapy may be boring, nonmotivational, and can generate a habituation process that limits the motor learning.^8,9^ There is a growing interest in finding therapeutic approaches, which provide a means of repetition and are highly motivating. Exercise-based computer games can facilitate high-volume complex task practice, enhance feedback of movement, and increase motivation of participants.^10^ Some bespoke systems and off-the-shelf commercial entertainment applications have emerged as new treatment approaches in rehabilitation training. Nevertheless, the cost and the requirement for special technical support of these systems limit the availability for most patients. Off-the-shelf commercial games are more affordable; however, those games are not specifically designed for therapeutic use and can be too difficult for children with physical and cognitive impairments.^11,12^ There is a need for feasible, effective, and low-cost, motion-controlled computer games for UE rehabilitation in children with CP.

The Microsoft Kinect™ is an infrared camera-based sensor a player can use to play a game through bodily movement without the need for handheld controllers. Moreover, the Kinect is inexpensive, portable, and simple to set up. The original Kinect design only identified joint displacement by measuring physical distance between joints. The distance can be very different for different individuals; it needs calibration when users change. Chang et al.^13^ made the Kinect design more robust by using angles instead of displacements of joints.

MIT's Scratch^14^ is widely used by learner developers to write games. Typically, Scratch games were controlled by a computer mouse or keyboard. A Scratch extension is a separate program that extends Scratch's capabilities, usually by connecting to external hardware devices. Howell developed Kinect2Scratch,^15^ which allows body tracking data from the Kinect be sent to Scratch. By using Kinect2Scratch, players can control Scratch games by moving their extremities.

The aims of this study were to (1) develop a suite of motion-controlled Kinect2Scratch games for UE training in children with CP and (2) investigate the feasibility and possible effects of these games. We hypothesized that the motion-controlled Kinect2Scratch games are feasible and effective for UE training in children with CP.

## Materials and Methods

### Game design

Considering the common problems of UE function in children with CP, and the motions that the Kinect sensor can detect, we chose two unilateral and one bilateral UE motion patterns (shoulder holding, reaching, and clapping hands) to play the games. The shoulder holding is the fundamental component of any UE movement. Reaching is a very common UE movement in ADL. Clapping hands is a bilateral coordinated movement. Three sample games (Table 1), “Hungry Shark,” “Alien Attack,” and “Hungry Ant,” developed by Howell, were chosen as the base game design. In “Hungry Shark,” the children needed to use their more affected UE in an “up and down” motion to control the shark. In “Hungry Ant,” the children used multidirectional reaching motion to control an ant. In “Alien Attack,” the children clapped their hands (using bilateral shoulder abduction–adduction motion) to launch missiles at the aliens.

**Table 1.** Characteristics of a Videogame for Health: (Kinect2Scratch Games)

	*Hungry Shark*	*Hungry Ant*	*Alien Attack*
Health topic(s)	Developing a suite of motion-controlled games for upper extremities training in children with cerebral palsy	Developing a suite of motion-controlled games for upper extremities training in children with cerebral palsy	Developing a suite of motion-controlled games for upper extremities training in children with cerebral palsy
Targeted age group(s)	Age between 5 and 15 years	Age between 5 and 15 years	Age between 5 and 15 years
Other targeted group characteristics	Clinical diagnosis of CP, involving at least one UE, manual ability graded as Manual Ability Classification System levels I–IV; had sufficient cognitive ability to play simple computer games; and vision sufficient to view the TV screen	Clinical diagnosis of CP, involving at least one UE, manual ability graded as Manual Ability Classification System levels I–IV; had sufficient cognitive ability to play simple computer games; and vision sufficient to view the TV screen	Clinical diagnosis of CP, involving at least one UE, manual ability graded as Manual Ability Classification System levels I–IV; had sufficient cognitive ability to play simple computer games; and vision sufficient to view the TV screen
Short description of game idea	Low cost, easily set up, user friendly, with therapeutic idea, motion-controlled games for UE training in children with CP	Low cost, easily set up, user friendly, with therapeutic idea, motion-controlled games for UE training in children with CP	Low cost, easily set up, user friendly, with therapeutic idea, motion-controlled games for UE training in children with CP
Target player(s)	Individual	Individual	Individual
Guiding knowledge or behavior change theory(ies), models, or conceptual framework(s)	Goal-directed, task-specific, and high-dose repetition therapy could lead to better motor outcomes. Exercise-based computer games can facilitate high-volume complex task practice, enhance feedback of movement, and increase motivation of participants	Goal-directed, task-specific, and high-dose repetition therapy could lead to better motor outcomes. Exercise-based computer games can facilitate high-volume complex task practice, enhance feedback of movement, and increase motivation of participants	Goal-directed, task-specific, and high-dose repetition therapy could lead to better motor outcomes. Exercise-based computer games can facilitate high-volume complex task practice, enhance feedback of movement, and increase motivation of participants
Intended health behavior changes	Improve UE function	Improve UE function	Improve UE function
Knowledge element(s) to be learned	The Kinect2Scratch videogames therapy is feasible and well accepted by children with CP These games could be a complementary strategy to conventional therapy	The Kinect2Scratch videogames therapy is feasible and well accepted by children with CP These games could be a complementary strategy to conventional therapy	The Kinect2Scratch videogames therapy is feasible and well accepted by children with CP These games could be a complementary strategy to conventional therapy
Behavior change procedure(s) (taken from Michie inventory) or therapeutic procedure(s) used	Functional repetitive movements in virtual environments are one of the principal mechanisms to favor the cortical reorganization and to eliciting neuroplastic changes in children with CP, videogames increasing the motivation of the player	Functional repetitive movements in virtual environments are one of the principal mechanisms to favor the cortical reorganization and to eliciting neuroplastic changes in children with CP, videogames increasing the motivation of the player	Functional repetitive movements in virtual environments are one of the principal mechanisms to favor the cortical reorganization and to eliciting neuroplastic changes in children with CP, videogames increasing the motivation of the player
Clinical or parental support needed? (please specify)	A supervised therapist or parents are needed to set up the system and instruct the children how to play the games and correct the inappropriate movement of children during the training sessions They also need to adjust the levels of difficulty throughout the training period by modifying target speed, movement accuracy, repetitions, and/or task complexity according to the performance of children	A supervised therapist or parents are needed to set up the system and instruct the children how to play the games and correct the inappropriate movement of children during the training sessions They also need to adjust the levels of difficulty throughout the training period by modifying target speed, movement accuracy, repetitions, and/or task complexity according to the performance of children	A supervised therapist or parents are needed to set up the system and instruct the children how to play the games and correct the inappropriate movement of children during the training sessions They also need to adjust the levels of difficulty throughout the training period by modifying target speed, movement accuracy, repetitions, and/or task complexity according to the performance of children
Data shared with parent or clinician	Yes	Yes	Yes
Type of game: (check all that apply)	Active, action	Active, action	Active, action
Story (if any)	None	None	None
Synopsis (including story arc)	None	None	None
How the story relates to targeted behavior change	None	None	None
Game components			
Player's game goal/objective(s)	Score on the screen/the shark eats the small fishes	Score on the screen/ant eats the number	Score on the screen/missile hits the alien
Rules	Increase score if the shark eats the small fishes	Increase score if the ant eats the number	Increase score if the missile hits the alien
Game mechanic(s)	The children need to use their more affected UE in an “up and down” motion to control the shark's movement to eat the coming fishes	The children used multidirectional reaching motion to control an ant to eat the numbers on the screen	The children did handclap (bilateral shoulder abduction–adduction) to launch missiles at the aliens
Procedures to generalize or transfer what's learned in the game to outside the game	Increasing shoulder holding ability, proximal stability, muscle power of shoulder and elbow, eye–hand coordination, reaction time, helping to use affected UE for reaching, holding in ADL	Increasing shoulder multidirectional movement, muscle power of shoulder and elbow, eye–hand coordination, reaction time, helping to use affected UE for reaching in ADL	Increasing bilateral coordination, eye–hand coordination, reaction time, shoulder holding, muscle power of bilateral shoulders and elbows, improving bilateral UE use in ADL
Virtual environment			
Setting (describe)	A TV screen, a Kinect, and a computer	A TV screen, a Kinect, and a computer	A TV screen, a Kinect, and a computer
Avatar			
Characteristics	There is no actual avatar, just a shark can be controlled	There is no actual avatar, just an ant can be controlled	There is no actual avatar, just missiles can be controlled
Abilities	A player used his or her more affected UE in an “up and down” motion to control the shark to eat the small fish	A player used multidirectional motion to control an ant to eat the number on the screen	A player needed to handclap (bilateral shoulder abduction–adduction) to launch missiles at the aliens
Game platform(s) needed to play the game	Kinect for Windows sensory device, a computer installed with Scratch visual programming language platform and Kinect2Scratch software	Kinect for Windows sensory device, a computer installed with Scratch visual programming language platform and Kinect2Scratch software	Kinect for Windows sensory device, a computer installed with Scratch visual programming language platform and Kinect2Scratch software
Sensors used	Kinect for Windows	Kinect for Windows	Kinect for Windows
Estimated play time	8 minutes	8 minutes	8 minutes

ADL, activities of daily living; CP, cerebral palsy; UE, upper extremity.

When playing the games, the children were asked to keep their elbows extended and trunk at midline. We used audio and text cues to prevent the children using inappropriate strategies, such as trunk deviation or elbow flexion, to play games. The acceptable range of trunk and UE movement was determined by therapists and stored in the games. If players exceeded the range, the game supplied an on-screen text reminder and audio prompt.

### Participants

We recruited children who received rehabilitation training in a tertiary hospital, had clinical diagnosis of CP, involving at least one UE, aged between 5 and 15 years, manual ability graded as Manual Ability Classification System levels I–IV; had sufficient cognitive ability to play simple computer games; and vision sufficient to view the TV screen to join this study. Children who had severe comorbidities, incapacity to sit even with an external support, surgical intervention in the year before study onset, botulinum injections in the 6 months before study onset, and noncontrolled epilepsy, were excluded.

The Ethics Committee of the CGMH approved the study. Written informed consent was obtained from the legal guardians of all participating children before being recruited to the study.

### Interventions

Each game play took 8 minutes, with a 2-minute break between every two games. The number of movements that the player made was not controlled. We suggested children complete 24 sessions for 12 weeks. Children sat or stood, depending on their ability, and this was kept constant across sessions.

A supervised occupational therapist instructed the children how to play the games and corrected the inappropriate movement of the children verbally or with manual guidance if the text or audio reminder did not work. The therapist adjusted the levels of difficulty of the games by modifying target speed, movement accuracy, repetitions, and/or task complexity according to the performance of the child. The therapist could also change the avatars in the games according to the child's preferences. For example, most children did not like the ant, it was replaced by a car.

Because our Kinect2Scratch games only focused on proximal UE motion training, we provided additional 30 minutes of conventional occupation therapy, focusing on hand function training, at each training session. The conventional occupational therapy was administered by another therapist. Treatment orders of Kinect2Scratch games and conventional occupation therapy were randomized.

### Outcome measures

This was a proof-of-concept feasibility study. Feasibility of Kinect2Scratch gaming training was the primary outcome of interest, including adherence, engagement, satisfaction, and safety.^16,17^ We also recorded the technical problems of delivery with the Kinect2Scratch games.

Adherence was represented by session attendance. We used the Pittsburgh Rehabilitation Participation Scale (PRPS)^18^ to assess the average degree of children's engagement in the first and last 2 weeks of either Kinect-based or conventional training according to the training records of the therapists. This assessment is a six-point scale of observed treatment engagement (effort and motivation). The PRPS had high inter-rater reliability (intraclass correlation coefficient [ICC] = 0.91 for occupational therapists, ICC = 0.96 for physical therapists).^18^ Satisfaction was evaluated at the end of the 12-week intervention. Children used 5-point Likert scales to rate the enjoyment of the Kinect2Scratch games training from “strongly not enjoyed” (1) to “strongly enjoyed” (5) and ranked the three games according to their preference. For children who did not understand the meaning of 5-point Likert scales, we used the bar chart to help the description of the 5-point Likert scales. The caregivers were interviewed to ask about the change of the children's UE function, children's motivation, and the general comments. About safety, we recorded any adverse events within the treatment sessions and during the study period.

Possible efficacy of Kinect2Scratch games was the secondary outcome of interest and was assessed at the beginning (T0) and after 24 sessions of training (T1) of the treatment. We used Quality of Upper Extremities Skills Test (QUEST)^19,20^ to assess the change of body function of the participants. Activity level was assessed by using the Box and Block Test (BBT),^21^ Melbourne Assessment 2 (MA2),^22^ and ABILHAND-kids score.^23^

QUEST evaluates four domains: upper limb dissociated movements, grasp function, protective upper limb extension, and weight bearing. The total score and each domain score were used for further analysis.

The BBT is a reliable and valid assessment tool of hand function.^21,24^ Children were asked to grasp small wooden cubes and move them from one side of the box to the other as fast as possible within 60 seconds.

The MA2 is a tool for evaluating quality of unilateral upper limb movement (movement range, accuracy, dexterity, and fluency) in children with neurological conditions aged from 2.5 to 15 years. It comprises 14 test items of reaching to, grasping, releasing, and manipulating simple objects.^22,25^

The QUEST and MA2 were recorded by video for scoring, scorers were blinded to the group allocation and the time point of assessments.

The ABILHAND-kids is a Rasch-derived, parent-completed questionnaire of 21 unilateral and bilateral activities. Each item is graded as easy, difficult, or impossible for the child to achieve.^26,27^

### Statistical analysis

The Statistical Package for Social Science (SPSS 12.0 for Windows; SPSS, Chicago, IL) was used for statistical analysis. Descriptive statistics were used to summarize baseline characteristics and feasibility data. We used the Shapiro–Wilk test to assess the normality of data distribution. Non-normally distributed data reported as median and interquartile range (IQR), and normally distributed data reported as mean ± SD in the text. Within-subject comparisons were examined using paired *t*-test for normally distributed data and with the Wilcoxon signed-rank test for non-normally distributed data. All significant tests were two tailed and differences were considered to be statistically significant at a *P*-value <0.05. Because this was a proof-of-concept study, no corrections for multiple testing were done.

## Results

Seventeen children with CP were screened for eligibility. Three patients were excluded because they did not meet the inclusion criteria, one changed her conventional rehabilitation training to another clinic, and thus, thirteen patients were included in the study. The mean age of participants was 6.9 ± 2.72 years, 6 (46.2%) with hemiplegia, 5 (38.5%) with diplegia, and 5 (38.5%) with Manual Ability Classification System level I–II (Table 2).

**Table 2.** Basic Characteristics of Participants (*N* = 13)

*Characteristics*	
Age, years	6.90 ± 2.72
Gender, boy	6 (46.2%)
Training side, right	9 (69.2%)
Education	
Preschool	7 (53.8%)
Primary school	6 (46.2%)
Type	
Hemiplegia	6 (46.2%)
Diplegia	5 (38.5%)
Quadriplegia	2 (15.4%)
MACS	
I–II	5 (38.5%)
III–VI	8 (61.5%)
GMFCS	
I–II	7 (53.8%)
III–V	6 (46.2%)

Values are presented as mean ± SD, or *N* (%).

GMFCS, Gross Motor Function Classification System; MACS, Manual Ability Classification System.

### Adherence

All children completed the 24 sessions of intervention. However, after 10–12 sessions of training, there were five children whose motivation in games training faded gradually. We needed to encourage them to complete the training during the late training course.

### Engagement

At the first 2 weeks of training, seven children had excellent, one very good, one good, three fair, and one poor participation in Kinect-based training. Two children had excellent, two very good, four good, and five fair participation in conventional rehabilitation. Children had a significantly higher level of participation in Kinect training than in conventional rehabilitation sessions [6 (3–6) vs. 4 (3–6) *P* = 0.04]. At the last 2 weeks of training, five children decreased their participation level from excellent to good or even fair in Kinect training, while two children raised their participation level from fair to good in Kinect training. The participation level in conventional rehabilitation was constant. There was no significant difference in participation between Kinect and conventional training sessions in late intervention course [4 (3–5) vs. 4 (3–6) *P* = 0.55].

### Satisfaction

In general, children and parents were satisfied with the system. Four (30.77%) children enjoyed, eight (61.54%) strongly enjoyed the games training, and the mean score of enjoyment was 4.54 ± 0.66. The children preferred the “Alien Attack” game most, followed by “Hungry Ant” game. The children complained that the motion to play the Hungry Shark game was difficult.

Parents reported that after intervention their child used the affected UE more frequently, could carry heavier objects, and had better quality of UE movement compared with the performance before intervention. Five parents reported that their child showed great enthusiasm in games at early stage of training, but the enjoyment of playing declined gradually. The possible reasons were (1) few games available, (2) little variation and lower quality of the graphics compared with commercial games, (3) lack of peer competition or rewards, and (4) the motor demands for gameplay were difficult for their child.

### Safety

No major adverse events occurred. Nine children complained of mild shoulder soreness at an early stage of intervention. Such soreness did not disrupt their training. After several sessions of intervention, those complaints subsided spontaneously.

### Technical problem

In general, the accuracy of motion detection of Kinect is acceptable, but we still experienced some technical joint tracking issues. The Kinect tracking algorithms work best with standing participants, and when all limbs are visible to the camera sensor. If a limb is partially occluded (e.g., an arm is hidden behind the subject's back), then the Kinect cannot determine the accurate position of the limb and will only track the visible limbs with a high degree of confidence. As some of the subjects were seated in the study, the Kinect may not track the hip and leg positions with accuracy.

The detection problem occurred most common with short stature children (the shortest child in our study was 95 cm tall) with poor seated positioning. The other condition for detection problems was when two joints overlapped in the camera. At the above conditions, the therapist usually could adjust the positioning of the Kinect sensor or the angle of inclination of the sensor to solve the problem.

Originally, we used auditory and text cues to remind the children that they had improper movement patterns, however, this was not effective. The children either ignored the reminder or became angry. We disabled the auditory and text cues in the later courses of training, and the therapist reminded children who had improper movement patterns verbally. For some children, the therapist needed to do hands-on correction for improper movement patterns.

### Efficacy

After intervention, the children had significant improvement in total QUEST score (median score preintervention 67.65, postintervention 75.84, *P* < 0.01), particularly in domains of dissociated movements (preintervention 76.56, postintervention 82.81, *P* < 0.01) and weight bearing (preintervention 88, postintervention 100, *P* < 0.01). There was significant improvement in MA2 (preintervention 52.31 ± 21.64, postintervention 58.85 ± 15.33, *P* = 0.02), the improvement mainly came from range motion (preintervention 16.77 ± 5.86, postintervention 18.54 ± 4.52, *P* = 0.05) and fluency (preintervention 9.85 ± 5.00, postintervention 11.54 ± 4.26, *P* = 0.01). There was no significant improvement in BBT (preintervention 12.31 ± 8.29, postintervention 13.85 ± 8.37, *P* = 0.07) and ABILHAND-kids score (logits preintervention 1.35 ± 1.90, postintervention 1.89 ± 2.31, *P* = 0.30) (Table 3).

**Table 3.** Outcome Measures at Baseline and Postintervention

	*Preintervention*	*Postintervention*	
*Outcome measures*	*Mean ± SD or median (IQR)*	*Mean ± SD or median (IQR)*	P
QUEST			
Total score	67.65 (56.57–82.54)	75.84 (67.15–86.78)	<0.01
Dissociated movement	76.56 (66.41–82.03)	82.81 (69.53–88.29)	<0.01
Grasp	45.59 ± 22.34	51.28 ± 20.28	0.06
Weight bearing	88 (74–98)	100 (87–100)	<0.01
Protective UL extension	64.74 ± 24.75	70.94 ± 25.97	0.08
Box and Block Test (number)	12.31 ± 8.29	13.85 ± 8.37	0.07
MA2			
Total score	52.31 ± 21.64	58.85 ± 15.33	0.02
ROM	16.77 ± 5.86	18.62 ± 4.52	0.05
Accuracy	18 (10.5–22)	19 (16–22.5)	0.09
Dexterity	9.23 ± 5.02	9.92 ± 3.8	0.24
Fluency	9.85 ± 5	11.54 ± 4.26	0.01
ABILHAND-kids (Logits)	1.35 ± 1.9	1.89 ± 2.31	0.30

Non-normally distributed data reported as median and IQR, and normally distributed data reported as mean ± SD.

IQR, interquartile range; MA2, Melbourne Assessment 2; QUEST, Quality of Upper Extremities Skills Test; ROM, range of motion; UL, upper limb.

## Discussion

Our study showed that 24 sessions of Kinect2Scratch videogames training for UE function in children with CP were feasible, and might be effective.

Even though we proved the feasibility of Kinect2Scratch games, there were some technical problems to be considered. Error-prone systems resulted quickly in frustration, reduced motivation, and decreased compliance. We experienced some movement detection problems. Although the problems were usually solved by the supervised therapist, it is important to be aware of the detection problems, especially for short stature children in a seated position. The second problem was that we used auditory and text cues to remind the children when they had improper movement patterns, however, the effect was poor and even made the children angry. Therefore, we do not suggest such cue use in future game design.

An important issue for successfully delivering the interventions for children with CP is the motivation. In our study, children had great motivation to play the games at the early stage of intervention, but the three simple games were insufficient to maintain the children's interest for a long time. Burdea et al.^28^ also reported that children with CP quickly lost interest in playing videogames when the games were no longer a challenge and new games were not available. To address this issue, we should create more games and improve the game design and the quality of the graphics. In addition, we thought if children's family and/or friends were able to play with them in competitive or collaborative ways, this might delay or prevent the faded motivation. Moreover, using adequate rewards, such as bonus, at the end of the game could probably be helpful.

This was a proof-of-concept study; a noncontrolled group was essential to understand whether the proposed therapy could provide positive results in UE function. After intervention, the children had improvements in quality and function of gross UE movements, but not in the fine motor and ADL function. Our results were well in line with the findings of Zoccolillo.^29^ They found using the Xbox with Kinect controller could improve upper limb motor skills in children with CP (shown by QUEST), but the conventional therapy remained superior for improving performances in manual ADL (shown by ABILHAND-kids score). As we did a combination therapy (Kinect2Scratch combined with conventional therapies), we could not identify the intervention effect coming from Kinect2Scratch or conventional or both therapies. However, the context and requirement of training tasks might explain these improvements observed in this pilot study. In our design, the games were all controlled by shoulder motion combining with elbow extension. The conventional therapy focused on hand function. We suspected the improvements in gross upper limb movements might be due to the Kinect2Scratch game training. Our Kinect2Scratch games did not offer ADL and fine motor manipulation training, and therefore, it was unsurprising that changes in fine motor and ADL measures did not occur.

Levac et al.^30^ suggested nine potential active ingredients of interactive computer play interventions, which might help children with neuromotor impairments to improve their motor skills: (1) opportunities for practice, (2) task specificity, (3) flexibility to individualize treatment parameters, (4) feedback, (5) social play equalization, (6) neuroplastic changes, (7) problem-solving, (8) motivation, and (9) role of a support person. The Kinect2Scratch games offer highly repetitive task-specific training (the movements trained within the games are similar to real-world movements), allow for tailoring of the training parameters to individual needs and the progress of training, and provide visual and/or auditory feedback on task performance. Children need to do problem-solving repeatedly because of the unpredictability and provision of obstacles in games. During the games intervention, the supervised therapist provided verbal feedback that may have contributed to outcomes. We suggested the above features make the Kinect2Scratch games a potentially viable tool to train UE motor skills for children with CP. In future research projects, we should have a robust study design to assess the isolated effect of the Kinect2Scratch game training.

You et al.^31^ indicated that functional repetitive movements in virtual environments are one of the principal mechanisms to favor cortical reorganization and to elicit neuroplastic changes in children with CP. It was reported that the limb movements performed during videogame-based therapy were about three times greater than those performed during conventional therapy.^29^ The motion-controlled videogames training system thus offers an important step toward achieving the number of repetition of exercises, appearing necessary to drive neuroplastic changes.^32^ Even if there are benefits of repetitions of selected tasks, we still need to notice the risk of repetitive injury. Providing adequate rest during the training session and using variable movement patterns to play games could prevent repetitive injuries. Under such training principles, we did not find any persistent or severe soft tissue injury problems during our study period.

### Limitation

Although this study is an important step on developing and evaluating the potential of Kinect2Scratch games as the UE rehabilitation treatment in children with CP, it has some limitations.

Since the study was designed as a proof-of-concept study in only 13 children without any control group, it is not possible to make any firm conclusions as to whether this way of training is more efficient in promoting functional benefits than other types of training. In addition, it was an adjunct study, the children also received conventional rehabilitation, a general conclusion cannot be extrapolated regarding the isolated efficacy of the gaming system. The limited number of games had affected motivation and probably limited the potential effectiveness. In the future, we should develop more games to increase the motivation and participation for children with CP to play the games. Because we used first generation of Kinect, we only could design games using shoulder and elbow for controlling. In the future, we should use the new generation of Kinect to design the interactive games with fine motor and virtual ADL training to increase the clinical application value.

Despite the above limitations, our study was important for proving the feasibility of clinical application of Kinect2Scratch games. Our results also could be a reference for future randomized controlled studies in larger populations of children to assess the true benefits of this gaming system.

## Conclusions

Our study suggested that the Kinect2Scratch games may be feasible and acceptable by children with CP, but further research is required using larger samples before conclusions can be drawn.
